# Functional Integrative Levels in the Human Interactome Recapitulate Organ Organization

**DOI:** 10.1371/journal.pone.0022051

**Published:** 2011-07-20

**Authors:** Ouissem Souiai, Emmanuelle Becker, Carlos Prieto, Alia Benkahla, Javier De Las Rivas, Christine Brun

**Affiliations:** 1 INSERM, U928, TAGC, Marseille, France; 2 Université de la Méditerranée, UMR-S928, Marseille, France; 3 Institut Pasteur, Tunis, Tunisia; 4 Bioinformatics and Functional Genomics Research Group, Cancer Research Center (IBMCC-CIC, CSIC-USAL), Salamanca, Spain; 5 CNRS, Marseille, France; University of Leuven, Belgium

## Abstract

Interactome networks represent sets of possible physical interactions between proteins. They lack spatio-temporal information by construction. However, the specialized functions of the differentiated cell types which are assembled into tissues or organs depend on the combinatorial arrangements of proteins and their physical interactions. Is tissue-specificity, therefore, encoded within the interactome? In order to address this question, we combined protein-protein interactions, expression data, functional annotations and interactome topology. We first identified a subnetwork formed exclusively of proteins whose interactions were observed in all tested tissues. These are mainly involved in housekeeping functions and are located at the topological center of the interactome. This ‘Largest Common Interactome Network’ represents a ‘functional interactome core’. Interestingly, two types of tissue-specific interactions are distinguished when considering function and network topology: tissue-specific interactions involved in regulatory and developmental functions are central whereas tissue-specific interactions involved in organ physiological functions are peripheral. Overall, the functional organization of the human interactome reflects several integrative levels of functions with housekeeping and regulatory tissue-specific functions at the center and physiological tissue-specific functions at the periphery. This gradient of functions recapitulates the organization of organs, from cells to organs. Given that several gradients have already been identified across interactomes, we propose that gradients may represent a general principle of protein-protein interaction network organization.

## Introduction

In metazoans, differentiation and ontogeny processes lead to the formation of differentiated tissues. Ultimately, these tissues, alone or in combination, constitute organs and ensure specific physiological functions. Deciphering the combinatorial arrangements of transcription factors that lead to tissue-specific gene expression can help explain the diversity of the differentiated tissues and the fundaments of their differences at a global scale [1], [2]. However, the combinatorial arrangements of proteins, other than transcription factors, also contribute to the diversity of the hundreds of different differentiated cell types. Therefore, studying the organization of the protein-protein interaction networks could reveal novel information insight on tissue diversity. Furthermore, tissue-specific interactions can occur between proteins which are not strictly tissue-specifically expressed [3], therefore reinforcing the relevance of considering tissue specificity from an interactome perspective.

Protein-protein interaction maps (or interactomes) are sets of interactions that have been experimentally identified using either high throughput technologies (such as large-scale two hybrid screens and affinity purifications mass spectrometry [4]–[14]) or regular low-scale experiments. They are assembled into large networks representing sets of possible biophysical interactions between the tested proteins. However, because of the experimental methods used to identify interactions, interactomes lack spatio-temporal information, thereby hindering any studies on specific biological contexts or conditions. This has been overcome by the integration of secondary data types such as (i) gene expression to identify different types of hubs and sub-network markers in cancer [15]–[18] and (ii) functional annotations to highlight context-specific interactions [19].

**Figure 1:**
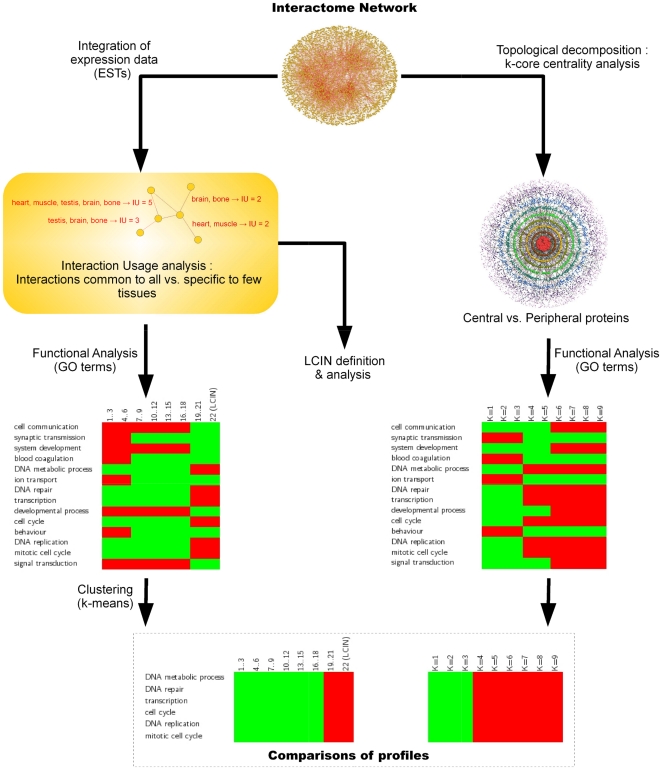
Workflow. The human interactome network is analyzed with two classification processes. **(Left side)** Using ESTs as source of gene expression, 22 tissular interactomes are inferred. The Interaction Usage of each interaction is determined, *i.e.* the number of tissues in which the interaction is possible given the co-expression of the partners in tissues. For each IU bin containing interactions with respect to their interaction usage, enrichment or depletion of GO Biological Process (BP) are computed and represented as a heatmap, which is ultimately clusterized using a k-means algorithm. **(Right side)** Following a k-core decomposition of the graph, enrichment or depletion of GO Biological Process are computed for proteins of coreness 1 to 9 and represented as a heatmap. **(Bottom)** For each UI cluster, enrichment/depletion profiles according to UI and topology are compared.

In the work described here (Figure 1), we aim at understanding the influence of function on interactome topology and answering questions such as ‘Does interactome topology reflect functional issues? Does function ‘shape’ the interactome? Is there an organizational ‘functional logic’ in the interactome?'. For this, we classified the interactions according to gene expression and the proteins according to network topology. By interpreting the results functionally using GO annotations, we address tissue diversity from an interactome point of view. We first defined the Largest Common Interactome Network (LCIN) which consists of those interactions possible in all the tissues tested, and whose proteins are mainly involved in housekeeping functions. We show that this LCIN corresponds to a ‘functional interactome core’, lying at the topological center of the interactome, and that tissue-specific interactions by definition excluded from the LCIN, are interestingly (i) centrally located when involved in regulatory and developmental functions and (ii) located at the periphery when involved in organ physiological functions. Combining interactions, expression, interactome network topology with cellular function annotations, we show that the organization of the interactome follows a functional gradient recapitulating the organization of organs.

## Results

### Inferring contextualized tissular interactomes

A human interactome composed of 27286 high confidence binary interactions between 9596 proteins was built by joining manually curated interactions derived from the literature, to those reported in APID [20]. First, using the EST clusters from the UniGene database [21] for gene expression data, we inferred several possible tissue-specific proteomes (Table S1). EST clusters were chosen as gene expression data rather than microarray data for coverage and function representation concerns. Indeed, in accordance with Zhu et al. [22] who have shown that microarray data exhibit a high rate of false negative, leading to an underestimation of the number of housekeeping genes, we confirmed the depletion of housekeeping gene detected in microarray data compared to ESTs (File S1).

Second, we postulated that if genes encoding two interactors are co-expressed in a given tissue, then that interaction is possible in that tissue. If either of the genes is not expressed in that tissue, it is assumed that the gene product is also absent, and therefore, that the interaction is impossible. In this way, we recovered 45 inferred proteomes and interactomes corresponding to the ‘body sites’ proposed by UniGene. We eliminated small and relatively incomplete interactomes due to poor EST coverage of certain tissue transcriptomes [22], and only considered the 22 largest contextualized interactomes, which contain more than 10 000 interactions, for further studies (Table S1).

### Interaction usage and Functions

#### Distribution of the ‘interaction usage’

A tissue-specific interaction may exist between proteins that are not necessarily tissue-specifically expressed. This is why it is different to investigate tissue-specific interactions rather than tissue-specific genes/proteins. Indeed, although the genes may be widely expressed, their shared tissular expression may be restricted to only few tissues, therefore leading to a tissue-specific interaction between gene products not tissue-specifically expressed. This accounts for 20% of the most tissue-specific interactions in our dataset (data not shown).

**Figure 2:**
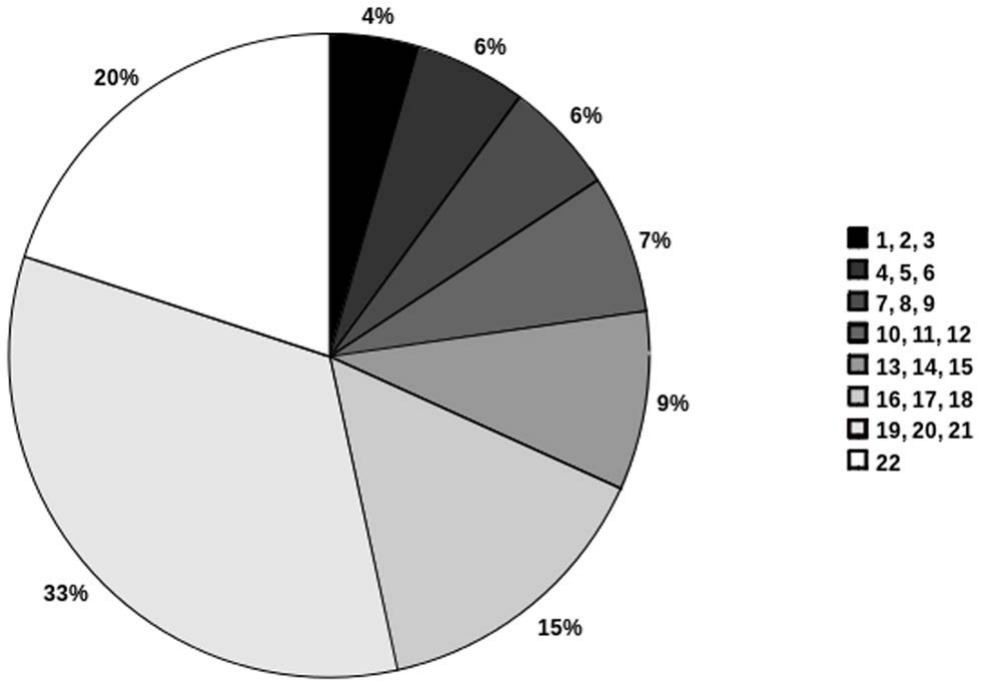
Distribution of the interaction usage in the human interactome. Bins correspond to the number of tissues in which interactions are possible.

To reflect this difference, we defined the notion of ‘interaction usage’ (IU) as the number of tissues in which an interaction is possible. This corresponds to the number of tissues in which both interactors are co-expressed (Table S1). The distribution of the IU values (Figure 2) shows first, the scarcity of strictly tissue-specific interactions: only 5% of the interactions are possible in less than 3 tissues and 11% in less than 6 tissues; second, that 77% of the interactions are possible in more than half of the tissues; and third, that 21% of the interactions are common to all the considered tissues.

The relevance of the IU distribution was assessed by showing that (i) it significantly differs from that obtained when randomly assigning proteins to tissue proteomes (Mood's median test, p-val = 0,03; Table S2); (ii) a similar distribution is obtained on a larger contextualized interactomes built from an interaction dataset of lower confidence (58189 interactions, 12531 proteins) (Table S3); (iii) it is in accordance with the human protein expression profiles recently revealed by immunological detection across a large number of cell types [23], in which only 2% of the tested proteins show a strict tissue-specificity and 20% are ubiquitously expressed (Table 1); (iv) it significantly differs from the distribution of gene/protein usage ( =  number of tissues in which the genes are expressed; Wilcoxon test, p-value <2.2e-16; (data not shown)). The validity of using EST information for proteome and interactome inferences is further validated by such agreement with experimentally derived protein localization data.

**Table 1:** Human protein expression profiles revealed by immunological detection [23] *vs.* inferred from ESTs.

	Human Protein Atlas [23] ^1^	This study ^2^
Nb investigated cell types / tissues	65	22
Method	Immunological detections	Data integration, inference from EST expression
% expressed proteins^1^/proteome^2^ (out of 4842^1^/17141 proteins^2^)	68 %	69%
Proteins commonly expressed (>60 cell types^1^/22 tissues^2^)	20%	20%
Proteins specifically expressed (<6 cell types^1^/1 tissue^2^)	3%	1.3%

#### The Largest Common Interactome Network: a functional interactome core devoted to housekeeping functions

The peculiarity of the distribution of the IU suggests a possible relationship between the usage of the interactions and the cellular functions they contribute to. We therefore investigated the interactions possible in all the studied tissues. They form the Largest Common Interactome Network (LCIN), which contains 4200 interactions between 1996 expressed proteins in all the tested tissues. This organization is not fortuitous, because when interactomes are inferred from randomly generated proteomes, none of the interactions are possible in all tested tissues, rendering the delineation of a LCIN impossible (Materials and Methods, Table S2).

**Figure 3:**
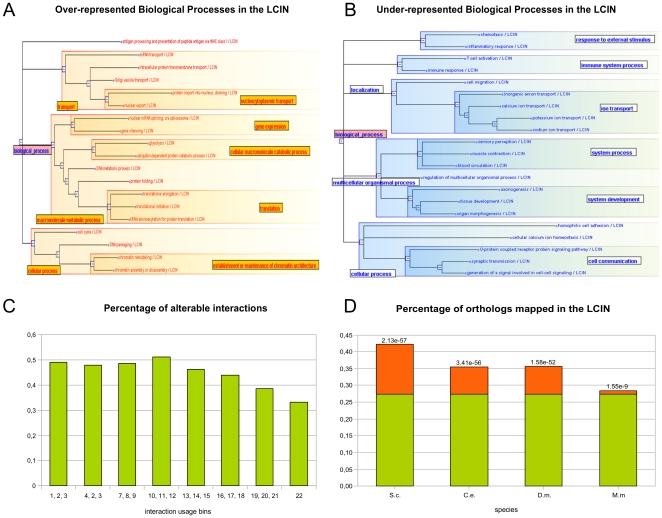
Largest Common Interactome Network analyses. (**A**) The sets of the most enriched (p-val<10^−5^) Biological Process (BP) annotations in the LCIN, visualized using SimCT [28] (http://tagc.univ-mrs.fr/SimCT/), a tool to visualize relationships between biological objects annotated to an ontology. (**B**) The sets of the most depleted (p-val<10^−5^) Biological Process (BP) annotations in the LCIN. (**C**) Distribution of interactions involving disease genes (according to OMIM) across the IU bins. (**D**) The LCIN is enriched in distant orthologues. Percentages of orthologs above the expected value (27%) are shown in orange. Enrichment p-values are given per organism.

Interactions of the LCIN are expected to participate in housekeeping cellular functions which occur in all cell types. Indeed, GO term analysis shows that the LCIN is particularly enriched (p-val<10^−5^) in proteins involved in nucleic acid and protein metabolic processes, intracellular transport, and cellular processes linked to cell cycle and nuclear organization. Conversely, interactions involved in organ morphogenesis, systems development and establishment (such as the nervous or immunological systems) or cell communication are particularly under-represented (p-val<10^−5^) in the LCIN (Figure 3A, 3B; Table S4). We believe, therefore, that the LCIN represents a functional interactome core devoted to housekeeping functions.

Additional features of the LCIN support this assertion. First, only 33% of the LCIN interactions involve disease genes (p-val = 1,73×10^−35^) compared to 43,5% in the rest of the interactome (cf. Material and Methods, Figure 3C), as expected from the observation that disease genes are generally expressed in a tissue-specific manner [24], [25]. Second, housekeeping proteins tend to be ‘ancient’ genes, highly conserved throughout evolution [26]. Accordingly, the LCIN shows a 1.3 to 1.5-fold enrichment in proteins having distant orthologues according to InParanoid (see Material and Methods, [27]): 35,5 to 42,2% of the orthologues found in *D. melanogaster, C. elegans* and *S. cerevisiae* belong to the LCIN (p-val_D.m_ = 1,58×10^−52^, p-val_C.e_ = 3,41×10^−56^, p-val_S.c_ = 2,13×10^−57^) (Figure 3D).

#### Interaction usage profiles according to cellular functions

**Figure 4:**
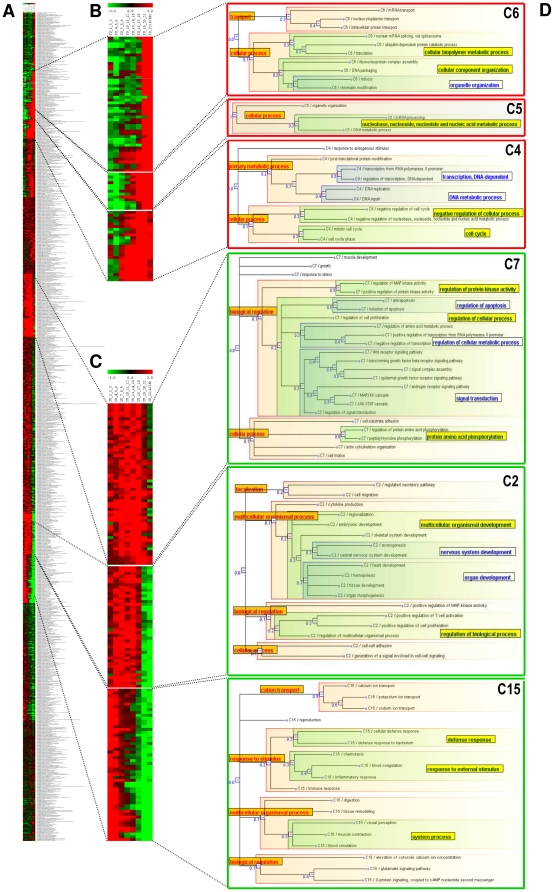
Interaction usage profiles according to cellular functions. (**A**) Heatmap representing the enrichment (in red)/depletion (in green) status of the 1023 GO Biological Process terms (lines) annotating the proteins participating in each IU bin (columns). (**B**) Representative clusters grouping GO terms highly enriched among common interactions and depleted in others. (**C**) Subtrees summarizing the relationships between GO terms grouped within clusters. For clarity, groups of tree branches are highlighted and annotated to the GO term corresponding to the deeper node of the subtree (framed GO terms). (**D**) Representative clusters of GO terms enriched among interactions possible in only a few tissues and depleted among common interactions. Dotted lines link clusters from the initial heatmap to the corresponding enlargements and GO term subtrees.

As shown above, the more common interactions are mainly involved in housekeeping functions. To investigate the remaining interactions, which should account for the functional and morphological differences between cells and tissues, we extended the previous LCIN analysis to the other IU categories (interactions binned according to the number of tissues in which they are possible; Figure 2). A heatmap representing the enrichment/depletion of GO Biological Process terms for the proteins in each IU category, is shown in Figure 4A (see Material and Methods). The observed enrichment/depletion profiles of the 1023 GO terms common to all IU categories were then grouped in 15 clusters using the k-means algorithm (6 clusters are detailed in Figure 4, the 9 others in Figure S1, S2, S3). The GO terms grouped in each cluster were represented using SimCT, a web-based tool that provides a simplified subgraph of the ontology, facilitating the interpretation of functional annotations [28]. Two main annotation profiles are distinguishable (Figure 4D): GO terms that are over-represented among proteins whose interactions were detected in many tissues and under-represented in the rest, and conversely, terms that are overrepresented among proteins whose interactions are possible in only few tissues and depleted in the rest. These annotation profiles are discretized by the classification process, leading to clusters into which the enrichment or the depletion status of terms progressively extends from the categories containing the more common interactions to the others.

Firstly, the clusters 6, 5 and 4 (Figure 4B) and 9, 10, 14 (Figure S1) group terms over-represented among the more common interactions (possible in 7 to 22 tissues according to the cluster) and depleted among interactions possible in only a few tissues. As expected and detailed in the previous paragraph, GO terms enriched solely in the more common interactions correspond to housekeeping functions (such as ‘DNA replication’, ‘DNA repair’, ‘mRNA processing’, ‘translation’ and ‘transport’, Figure 4D). Interestingly, in addition to terms related to housekeeping functions, cluster 4 also contains terms referring to the regulation of housekeeping functions (as ‘regulation of cell cycle’). Unlike interactions mediating the housekeeping functions themselves, interactions regulating these processes are not expected to be shared by all tissue types but to be more tissue-specific. Indeed, cluster 4 contains terms over-expressed in all tissues as expected for housekeeping interactions, as well as in a more restrained number of tissues, as expected from regulatory interactions. This cluster may illustrate the specificity of the regulation of common processes in particular tissues.

Second, clusters 7, 2, and 15 (Figure 4C) and 1, 3, 8, 11, and 12 (Figure S2, S3) group terms depleted among the more common interactions and over-represented elsewhere. These terms (Figure 4D) are related to regulation and signal transduction (such as ‘regulation of MAP kinase activity’, ‘Wnt receptor signaling pathway’ or ‘EGFR signaling pathway’ in cluster 7), organ development (as ‘central nervous system development’, ‘heart development’ or ‘hemopoiesis’ in cluster 2), and the physiological functions of the organs (for instance ‘muscle contraction’, ‘blood circulation’ or ‘visual perception’ in cluster 15). Therefore, regulatory and physiological processes appear to be excluded from the more common interactions, therefore mediated by tissue-specific interactions.

### Functions, Interaction Usage and Network Topology

**Figure 5:**
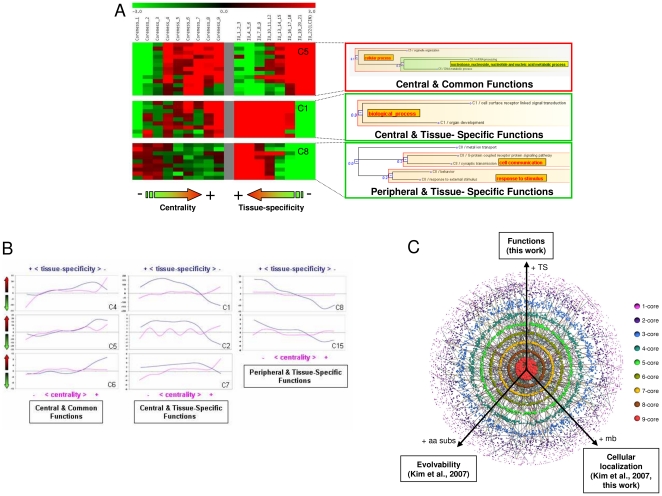
Interaction usage, cellular functions and interactome topology. (**A**) Comparison of the heatmaps of enrichment/depletion of GO terms annotating the proteins of each IU bin (middle panel) and each *k-*core category (left panel). The right panel represents the subtrees summarizing the relationships between GO terms grouped within the shown clusters. (**B**) The tendency of each cluster according to each criterion (topology in pink and interaction usage in blue) is visualized by transforming the juxtaposed heatmap representation into a graph in which each IU and *k-*core category is represented by its median value. (**C**) Gradients as a trend of interactome organization. Interactome layers corresponding to the *k-*core categories of the graph are visualized using the Caida tool [42]. Proteins of *k-*core 9 are red, *k-*core 8 are brown, *k-*core 7 are yellow, etc. For clarity, only 10% of the graph edges are shown.

Intuitively, as the LCIN forms the core cellular machinery common to all tissues, it is tempting to speculate that it should be buried in the innermost part of the interactome, leaving the topological periphery of the interactome to more tissue-specific functions. To verify this hypothesis, we used the *k-*core decomposition of the graph to define the topological layers of the interactome [29]. Essentially, this means progressively pruning the graph vertices (proteins) according to the number of edges (interactions) linking them to the connected component [30]. Proteins of high *k-*core are topologically central in the network whereas proteins of low k-core are peripheral. In the studied interactome, proteins of the highest k-core (*k-*core 9) are almost double what would be expected by chance in the LCIN (p-val = 3,78×10^−14^), indicating a correlation between the centrality of a protein and its involvement in common interactions. By extension, proteins of the highest *k-*core should be involved in housekeeping functions. Building on this idea, we addressed the possible relationship between the IU, network topology and function. As before, we calculated over- and under-representation of Gene Ontology terms annotating the proteins in each *k-*core category. The resulting data, for each of the clusters previously defined according to the IU categories, are shown as heatmaps and graphs in Figure 5 and Figures S4, S5, S6.

#### Housekeeping functions are common to all tissues and topologically central

Functions that are over-represented in the LCIN and therefore mediated by the more common interactions are also enriched among the high *k-*core proteins and depleted in the low *k-*core ones (cluster 4, 5, 6 on Figure 5, and 9, 10 on Figure S4). In this case, the enrichment/depletion profiles of the GO terms show the same tendency with respect to the IU and the topology. Housekeeping functions and their regulation are therefore mediated by interactions present in a large number of tissues (from 7 to 22) and are centrally located in the interactome. Thus, we show that all these interactions, including those of the functional core previously defined, map to the topological center of the interactome.

#### Regulatory and developmental functions are tissue-specific and topologically central

Interestingly, other functions, although depleted among the more common interactions, are found enriched among the more central proteins of the interactome (clusters 1, 2, 3, 7 and 11, Figure 5, Figure S5), as shown by the inversion of their enrichment/depletion profiles in the more central and common parts of the interactome. This suggests that these functions, although almost excluded from the functional core, are mediated by topologically central interactions. More particularly, they correspond to the regulation of biological and cellular processes (such as ‘regulation of apoptosis’, ‘positive regulation of T cell activation’) and the molecular regulatory processes themselves (for instance, ‘signal transduction’ or ‘protein amino acid phosphorylation’) (Figure 5). They are also related to the developmental processes of the organs and organisms (such as ‘regionalization’, ‘embryonic development’, ‘organ morphogenesis’).

#### Physiological functions are tissue-specific and topologically peripheral

Finally, a third type of functions mediated by tissue-specific interactions is found relatively enriched among proteins of the lowest *k-*core (clusters 8, 12 and 15) (Figure 5, Figure S6). These tissue-specific interactions are related to physiological processes and their underlying molecular processes (such as ‘cognition’, ‘spermatogenesis’, ‘synaptic transmission’ or ‘cation transport’) and lie at the periphery of the interactome.

It appears that the tissue-specific interactions excluded from the functional core are located at the center as well as at the periphery of the interactome, each location corresponding primarily to a particular type of tissue-specific function. The topological criterion, therefore, distinguishes between a tissue-specificity related to the regulatory and the developmental processes on one hand and the physiological processes on the other.

## Discussion

Tissue-specific interactions can occur between proteins that are not necessarily tissue-specifically expressed [3]. Indeed, a tissue-specific function can be performed by proteins that, while not exclusive to the tissue of interest, can only interact in that tissue. This explains why considering tissue-specific interactions rather than proteins should, therefore, bring a deeper functional insight to the understanding of cell and tissue diversity. We adopted this point of view to investigate whether the topology of the interactome reflects functional issues. To do so, we classified interactions according to gene expression on one hand, proteins according to their network topological features on the other hand, aiming to finally interpret the results in the light of functional annotations. The novelty of the approach relies on the common functional interpretation of two independent classification schemes. This allowed distinguishing two types of tissue-specific interactions which would not have been detected otherwise, by a global interactome study.

Classifying interactions by integrating gene expression allows estimating their level of tissue-specificity. Although proteomic data would have been the data of choice for protein tissue expression, such comprehensive data are not yet available to our knowledge. We then chose EST as tissular gene expression source to contextualize protein interaction rather than microarray data mainly for two reasons: interactome coverage and protein function representation. Indeed, the coverage of the used expression data is a concern because the studied interactome is only a subset of limited size. As shown in File S1 and in accordance with Zhu et al. [22], the number of housekeeping genes detected with microarrays (taken from Gene Expression Atlas [31] in this study and from [32] in [22]) is underestimated when compared to EST and immuno-detected proteins. This fact is probably due to the stringent thresholds chosen for microarray analyses. Moreover, the use of microarray data for contextualization and the consequent high rate of false negative among housekeeping genes would (i) trivially lead to very small tissular interactomes and more importantly, (ii) introduce a bias in the functional analyses of the inferred interactomes, ie. a depletion of interactions occurring between housekeeping gene products and tissue-specifically expressed proteins.

Centrally located tissue-specific interactions mediate regulatory and developmental functions while peripheral ones carry out physiological functions. This appears illustrating the differences between, for instance, the tissue-specificity of (i) the regulatory interactions that can occur between regulators and housekeeping proteins and lead to tissue-specific transcriptional activation of gene expression, and (ii) the interactions responsible of the assembly of tissue-specific protein complexes involved in physiological functions.

With regards to housekeeping interactions, this work defines an interactome functional core present in all the investigated tissues and essentially formed by interactions devoted to ubiquitous functions. This centrality of the housekeeping functions is reminiscent of the organization evoked for unicellular organisms by Vinogradov [33], while studying the modularity of the yeast interactome. Noticeably, this is, to the best of our knowledge, the first time that such evidence emerges from the interactome analysis of a metazoan organism, suggesting that such organization may be a trend of the functional interactome organization across species.

Bossi and Lehner [3] demonstrated that housekeeping proteins interact with tissue-specifically expressed proteins, without providing functional insights on these particular interactions. Our analysis interestingly extends their results by showing that some of those interactions are regulatory. Specifically, the annotation ‘intracellular signaling cascade’ is the most over-represented (p-val = 2.36×10^−8^, data not shown) among those proteins of the functional core that interact with tissue-specifically expressed proteins. Finally, regulatory interactions appear as topologically central, irrespective of the biological process considered (housekeeping or tissue-specific).

Together, these results led to the proposal that the functional core subnetwork formed by the common interactions combined with the tissue-specific regulatory interactions corresponds to the topological center of the interactome. Interestingly, this particular organization may be a hallmark of the metazoan interactome since noticeably, the topologically central part of the yeast interactome has been previously demonstrated to be rather enriched in evolutionary conserved and essential proteins [29] mainly dedicated to housekeeping functions [33].

Tissue-specific interactions involved in physiological functions are found at the periphery of the interactome. Noticeably, this links the observations that tissue-specific proteins are more likely to be recent evolutionary innovations [34], that rapidly evolving genes are expressed in a narrow range of tissues [35] and that proteins having the higher potential to evolve are located at the interactome periphery [36].

The functional organization of the human interactome deciphered in this work recapitulates the integrative organization of organs, from cells to organ, by following a gradient of functions from center to periphery (Figure 5C). This reflects the fact that tissues and organs first acquire their specificity from the developmental and regulatory programs which build on common molecular mechanisms. Tissues and organs then become fully functional when physiological functions are established. Our results suggest that the pace of these events may be encoded in the organization of the interactome. Interestingly, this gradient is reminiscent of two other gradients recently described in interactomes: a gradient of evolvability suggested by the observation of the preferential peripheral location of human proteins under positive selection, and a gradient of cellular localization reflecting that proteins located at the cellular periphery are also peripheral in the network ([36] and data not shown). Gradients (such as of functions, evolvability or cellular location) may therefore represent a trend of protein-protein interaction network organization.

## Materials and Methods

### Protein-protein interaction datasets

A high confidence dataset of 27286 binary interactions involving 9596 proteins was built by joining (i) 2325 human interactions manually extracted from the literature and (ii) 24961 binary interactions compiled in APID [20] (Table S1). Interactions identified by at least one experimental method leading to the detection of binary interactions (Table S5) were selected. A larger dataset of lower confidence (comprising 58189 interactions between 12531 proteins) was built by adding the 2325 manually curated interactions to the complete set of human interactions present in APID.

### Expression data, proteome and interactome inferences

Clusters of expressed sequence tags (ESTs) from the UniGene database (Homo sapiens release 214 - June 23rd , 2008) [21] were used as source of gene expression data. For each cluster, a list of tissues in which ESTs were expressed was created, independently of their level of expression. After mapping their corresponding gene names to protein names by parsing UniProt files, the composition of 45 proteomes was inferred. Assuming that an interaction present in the interaction dataset is possible in a given tissue if both protein partners are expressed in this tissue, 22 tissular interactomes containing more than 10000 interactions each were inferred (Table S1) and used for further studies. Globally, 21010 interactions between 7293 proteins were found possible in at least 1/22 tissues. They formed the Largest Possible Interactome Network (LPIN).

### Interaction Usage

For each protein *a*, the expression profile is encoded by a vector *ve(a)* of 22 Boolean values where (1) represents the presence and (0) the absence of protein *a* in each of the 22 tissues. Formally, we define the interaction usage *w(a,b)* between protein *a* and protein *b* as the scalar product of the expression vectors *w(a,b)  =  ve(a).ve(b).* Intuitively, *w(a,b)* represents the number of tissues in which the interaction between *a* and *b* is possible.

### Functional Analyses, Heatmaps and Clusters

The functional enrichment/depletion of the LCIN was determined by comparing the Gene Ontology [37] annotations of the proteins involved in the LCIN to those involved in the LPIN using the GOToolBox application [38], which uses an hyper-geometric law corrected for multiple testing. All three ontologies were tested (Table S4).

This functional analysis was extended to the other IU bins (containing interactions with respect to the number of tissues in which they are possible) to reveal enriched or depleted GO Biological Process (BP) annotations in each bin. On average, 88% of the proteins per bin are annotated. Only GO terms annotating more than 10 proteins of the LPIN are considered in the analysis (Table S6). As a result, a *p-value(go,b)* is computed for each annotation *go* in each bin *b*. A global matrix *M* in which the lines are the GO BP terms and the columns are the IU bins is built. The matrix contains 1023 GO BP terms annotating the LPIN proteins. For each GO annotation *go* and for each bin *b*, the matrix *M[go,b]* contains *−log(p-value(go,b))* if annotation *go* is enriched in bin *b* and *log(p-value(go,b))* if it is depleted.

Heatmap visualization and further analyses were performed using MeV [39]. A k-means clustering algorithm was applied and the matrix was split into 15 clusters. The number of clusters was chosen following a Figure Of Merit (FOM) analysis which estimates the predictive power of a clustering algorithm [40].

### Centrality analysis and *k-*core decomposition

The interactome can be formally represented by a graph *G = (V,E)*, where *V* is the set of vertices and *E* the set of edges. The *k-*core decomposition of the graph [30], can be intuitively seen as a peeling process. A subgraph H(C, E|C) induced by the set *C* 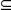 *V* is a *k-*core or a core of order k if and only if 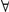 *v* 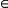 *C : degree_H_(v)* 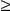 *k*, and H is the maximum subgraph with this category. A vertex *v* has a coreness *c* if it belongs to the *c-core* but not to the *(c+1)-core.* The algorithm used to compute the coreness was extracted from [41].

To explore whether the 15 functional clusters tend to have a clear behavior in terms of centrality, the enrichment or depletion of these 1023 annotations was computed for proteins of coreness 1 to 9. As previously explained, the p-values were considered negative if the annotation is under- and positive if the annotation is over-represented. These centrality values were not used for the clustering, but added subsequently.

## Supporting Information

Figure S1 **Common functions. Interaction usage heatmaps and SimCity trees for cluster 9, 14 and 10.**
(TIF)

Figure S2 **Tissue-specific functions. Interaction usage heatmaps and SimCity trees for cluster 1, 3 and 8.**
(TIF)

Figure S3 **Tissue-specific functions. Interaction usage heatmaps and SimCity trees for cluster 11 and 12.**
(TIF)

Figure S4 **Central and common functions.** ***K-*** **core and interaction usage heatmaps for cluster 4, 5, 6, 9 and 10.**
(TIF)

Figure S5 **Central and tissue-specific functions.** *K-*core and interaction usage heatmaps for cluster 1, 2, 3, 7 and 11.
(TIF)

Figure S6 **Peripheral and tissue-specific functions.** *K-*core and interaction usage heatmaps for cluster 8, 12 and 15.
(TIF)

Table S1 **Proteomes and Interactomes.** **(Sheet Proteins)** Composition of the 22 tissue-specific proteomes. For each protein, a binary vector indicates if the protein is absent (0) or present (1) in each tissue. The number of tissues within which the protein is considered as present is reported in the last column (#T). **(Sheet Interactions)** Composition of the 22 tissue-specific interactomes. For each interaction, the first value indicates the interaction usage, and then a binary vector indicates if the interaction is possible (1) or not possible (0) in each tissue.
(XLS)

Table S2 **Randomizations and Larger Interactome(Sheet Randomizations)** Observed vs. Randomized distribution of the interaction usage. 9 randomizations were performed. For each randomization, 22 proteomes respecting the sizes of the 22 observed proteomes were selected randomly, thus leading to 22 random tissue-specific interactomes. Then, the interaction usage of each interaction was computed. On average, the number of interactions in the LCIN is null in the randomizations (mean 0.1, standard deviation 0.3). **(Sheet Larger Interactome)** High quality vs. larger human interactome Interaction Usage distributions.
(XLS)

Table S3 **Interactome Features.**
(XLS)

Table S4 **Functional Analyses of the LCIN using the three ontologies of GO.** Terms of the Biological Process sub-ontology that are found over-represented (Sheet Enriched BP annotations) and depleted (Sheet Depleted BP annotations) among the protein annotations composing the LCIN relatively to the proteins composing the LPIN. Results are obtained with GOToolBox, using an hypergeometric law corrected for multiple testing with the Benjamini & Hochberg correction. The terms whose over/under-representation p-value is less than 1E-05 (in red/blue) have been used to build the SimCT trees (Figure 3). The same analyses have been performed for the two other sub-ontologies.
(XLS)

Table S5 **List of the experimental techniques and their PSI-MI considered as identifying binary interactions.**
(XLS)

Table S6 **Matrix.** Matrix representing the enrichment or the depletion of 1073 Biological Process (BP) annotations in different interaction usage bins, using the LPIN as the reference dataset. For each annotation, the column NB indicates the abundance of its instances in the reference dataset. Enrichment is represented by positive values: the greater the more enriched. Depletion is represented by negative values: the lower the more depleted. An help sheet is provided.
(XLS)

File S1 **Choosing expression data to contextualize interactome: ESTs vs. Microarrays.**
(DOC)

## References

[1] Zinzen RP, Girardot C, Gagneur J, Braun M, Furlong EE (2009). Combinatorial binding predicts spatio-temporal cis-regulatory activity.. Nature.

[2] Yu X, Lin J, Zack DJ, Qian J (2006). Computational analysis of tissue-specific combinatorial gene regulation: predicting interaction between transcription factors in human tissues.. Nucleic Acids Res.

[3] Bossi A, Lehner B (2009). Tissue specificity and the human protein interaction network.. Mol Syst Biol.

[4] Formstecher E, Aresta S, Collura V, Hamburger A, Meil A (2005). Protein interaction mapping: a Drosophila case study.. Genome Res.

[5] Gavin AC, Bosche M, Krause R, Grandi P, Marzioch M (2002). Functional organization of the yeast proteome by systematic analysis of protein complexes.. Nature.

[6] Gavin AC, Aloy P, Grandi P, Krause R, Boesche M (2006). Proteome survey reveals modularity of the yeast cell machinery.. Nature.

[7] Giot L, Bader JS, Brouwer C, Chaudhuri A, Kuang B (2003). A protein interaction map of Drosophila melanogaster.. Science.

[8] Ho Y, Gruhler A, Heilbut A, Bader GD, Moore L (2002). Systematic identification of protein complexes in Saccharomyces cerevisiae by mass spectrometry.. Nature.

[9] Ito T, Chiba T, Ozawa R, Yoshida M, Hattori M (2001). A comprehensive two-hybrid analysis to explore the yeast protein interactome.. Proc Natl Acad Sci U S A.

[10] Krogan NJ, Cagney G, Yu H, Zhong G, Guo X (2006). Global landscape of protein complexes in the yeast Saccharomyces cerevisiae.. Nature.

[11] Li S, Armstrong CM, Bertin N, Ge H, Milstein S (2004). A map of the interactome network of the metazoan C. elegans.. Science.

[12] Rual JF, Venkatesan K, Hao T, Hirozane-Kishikawa T, Dricot A (2005). Towards a proteome-scale map of the human protein-protein interaction network.. Nature.

[13] Stelzl U, Worm U, Lalowski M, Haenig C, Brembeck FH (2005). A human protein-protein interaction network: a resource for annotating the proteome.. Cell.

[14] Uetz P, Giot L, Cagney G, Mansfield TA, Judson RS (2000). A comprehensive analysis of protein-protein interactions in Saccharomyces cerevisiae.. Nature.

[15] Agarwal S, Deane CM, Porter MA, Jones NS (2010). Revisiting date and party hubs: novel approaches to role assignment in protein interaction networks. PLoS Comput.. Biol.

[16] Chuang H-Y, Lee E, Liu Y-T, Lee D, Ideker T (2007). Network-based classification of breast cancer metastasis. Mol. Syst.. Biol.

[17] Han JD, Bertin N, Hao T, Goldberg DS, Berriz GF (2004). Evidence for dynamically organized modularity in the yeast protein-protein interaction network.. Nature.

[18] Taylor IW, Linding R, Warde-Farley D, Liu Y, Pesquita C (2009). Dynamic modularity in protein interaction networks predicts breast cancer outcome. Nat.. Biotechnol.

[19] Rachlin J, Cohen DD, Cantor C, Kasif S (2006). Biological context networks: a mosaic view of the interactome.. Mol Syst Biol.

[20] Prieto C, De Las Rivas J (2006). APID: Agile Protein Interaction DataAnalyzer.. Nucleic Acids Res.

[21] Wheeler DL, Church DM, Federhen S, Lash AE, Madden TL (2003). Database resources of the National Center for Biotechnology.. Nucleic Acids Res.

[22] Zhu J, He F, Song S, Wang J, Yu J (2008). How many human genes can be defined as housekeeping with current expression data?. BMC Genomics.

[23] Ponten F, Gry M, Fagerberg L, Lundberg E, Asplund A (2009). A global view of protein expression in human cells, tissues, and organs.. Mol Syst Biol.

[24] Goh KI, Cusick ME, Valle D, Childs B, Vidal M (2007). The human disease network.. Proc Natl Acad Sci U S A.

[25] Lage K, Hansen NT, Karlberg EO, Eklund AC, Roque FS (2008). A large-scale analysis of tissue-specific pathology and gene expression of human disease genes and complexes.. Proc Natl Acad Sci U S A.

[26] Duret L, Mouchiroud D (2000). Determinants of substitution rates in mammalian genes: expression pattern affects selection intensity but not mutation rate.. Mol Biol Evol.

[27] Ostlund G, Schmitt T, Forslund K, Köstler T, Messina DN (2010). InParanoid 7: new algorithms and tools for eukaryotic orthology analysis.. Nucleic Acids Res.

[28] Herrmann C, Berard S, Tichit L (2009). SimCT: a generic tool to visualize ontology-based relationships for biological objects.. Bioinformatics.

[29] Wuchty S, Almaas E (2005). Peeling the yeast protein network.. Proteomics.

[30] Seidman SB (1983). Network structure and minimum degree.. Social Networks.

[31] Kapushesky M, Emam I, Holloway E, Kurnosov P, Zorin A (2010). Gene expression atlas at the European bioinformatics institute.. Nucleic Acids Res.

[32] Su AI, Wiltshire T, Batalov S, Lapp H, Ching KA (2004). A gene atlas of the mouse and human protein-encoding transcriptomes.. Proc Natl Acad SciU S A.

[33] Vinogradov AE (2008). Modularity of cellular networks shows general center-periphery polarization.. Bioinformatics.

[34] Lehner B, Fraser AG (2004). Protein domains enriched in mammalian tissue-specific or widely expressed genes.. Trends Genet.

[35] Park SG, Choi SS (2010). Expression breadth and expression abundance behave differently in correlations with evolutionary rates. BMC Evol.. Biol.

[36] Kim PM, Korbel JO, Gerstein MB (2007). Positive selection at the protein network periphery: evaluation in terms of structural constraints and cellular context.. Proc Natl Acad Sci U S A.

[37] Ashburner M, Ball CA, Blake JA, Botstein D, Butler H (2000). Gene ontology: tool for the unification of biology. The Gene Ontology Consortium.. Nat Genet.

[38] Martin D, Brun C, Remy E, Mouren P, Thieffry D (2004). GOToolBox: functional analysis of gene datasets based on Gene Ontology.. Genome Biol.

[39] Saeed AI, Bhagabati NK, Braisted JC, Liang W, Sharov V (2006). TM4 microarray software suite. Meth.. Enzymol.

[40] Yeung KY, Haynor DR, Ruzzo WL (2001). Validating clustering for gene expression data.. Bioinformatics.

[41] Batagelj V, Zaversnik M (2003). An O(m) Algorithm for Cores Decomposition of Networks.. http://arxiv.org/abs/cs/0310049.

[42] Alvarez-Hamelin J, Dall'asta L, Barrat A, Vespignani A (2005). k-core decomposition: a tool for the visualization of large scale networks.. http://arxiv.org/abs/cs/0504107.

