# Supplementary figures and images for: Functional Integrative Levels in the Human Interactome Recapitulate Organ Organization
Source: PLoS One. 2011 Jul 20;6(7):e22051. doi: 10.1371/journal.pone.0022051 (PMC3140469; doi:10.1371/journal.pone.0022051)

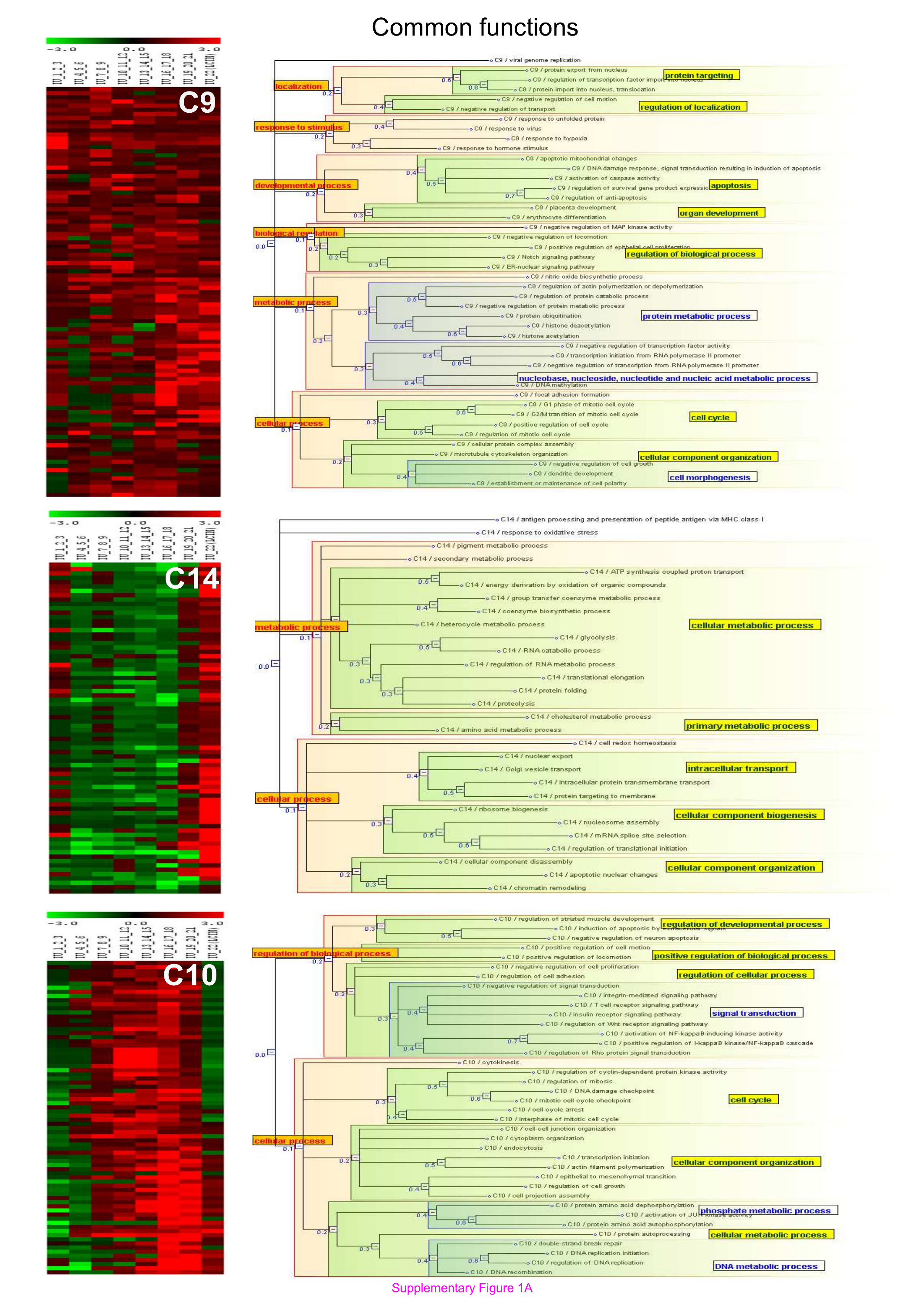

Supplement: Figure S1 — Common functions. Interaction usage heatmaps and SimCity trees for cluster 9, 14 and 10. (TIF) [file pone.0022051.s001.tif]

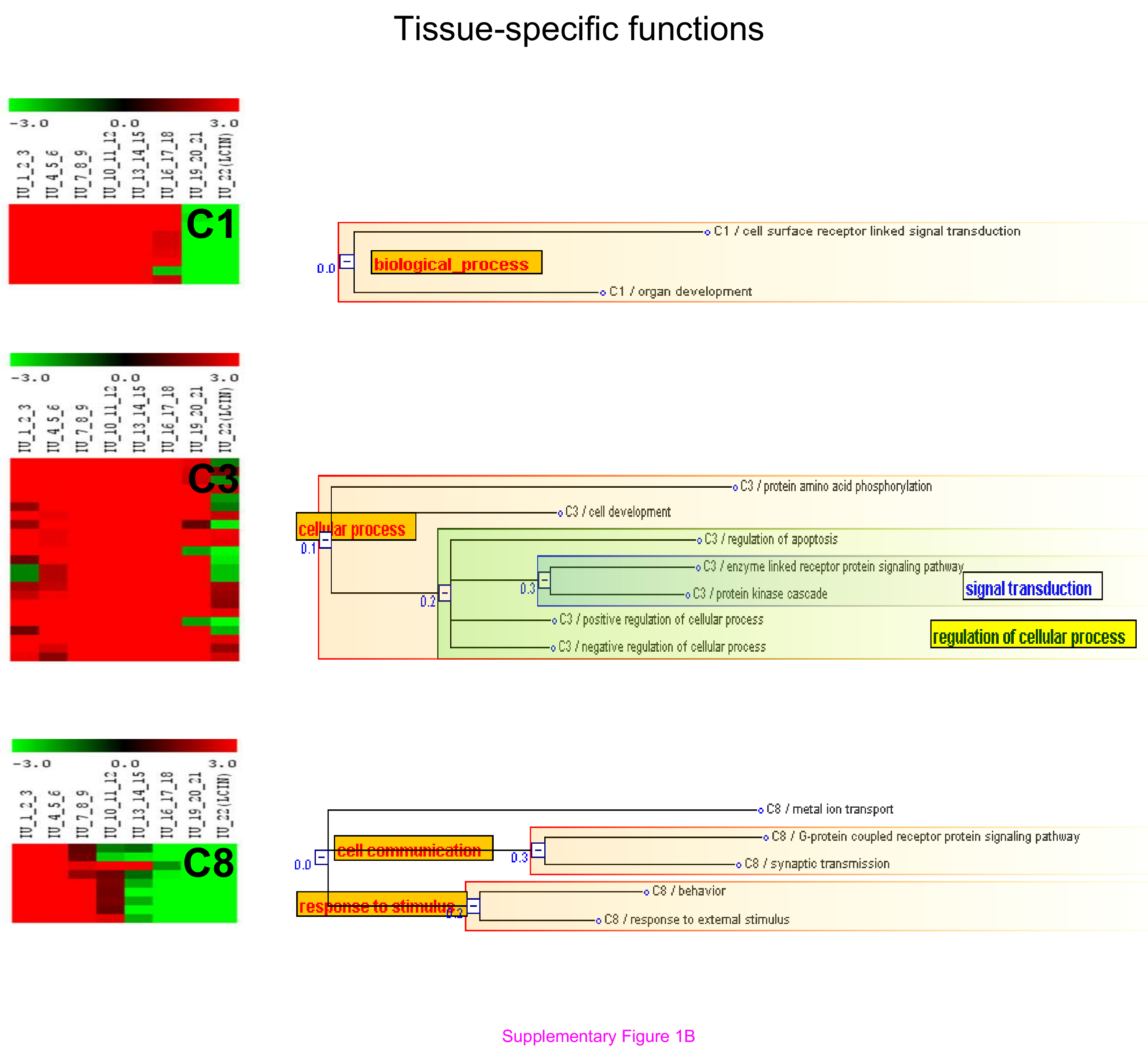

Supplement: Figure S2 — Tissue-specific functions. Interaction usage heatmaps and SimCity trees for cluster 1, 3 and 8. (TIF) [file pone.0022051.s002.tif]

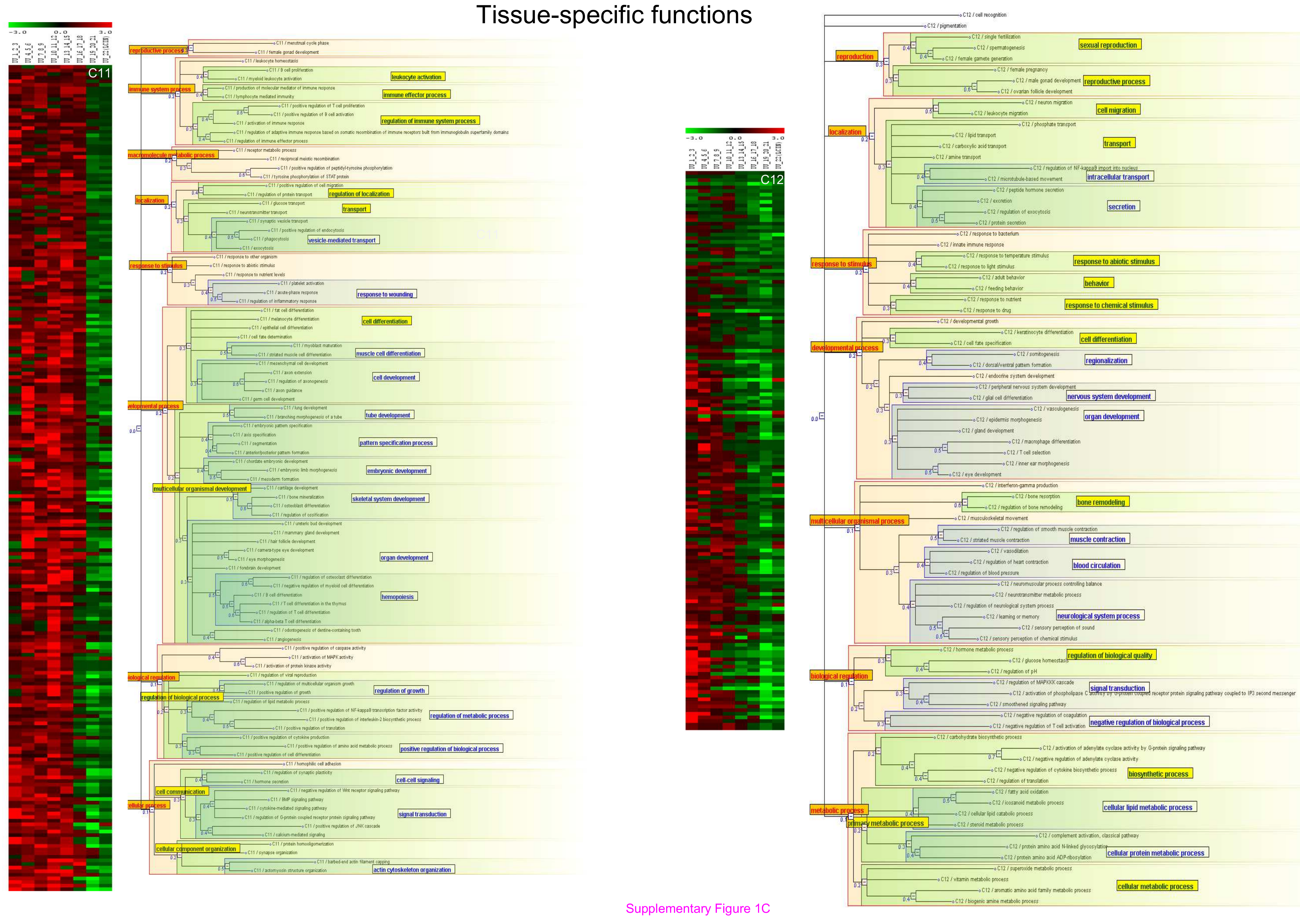

Supplement: Figure S3 — Tissue-specific functions. Interaction usage heatmaps and SimCity trees for cluster 11 and 12. (TIF) [file pone.0022051.s003.tif]

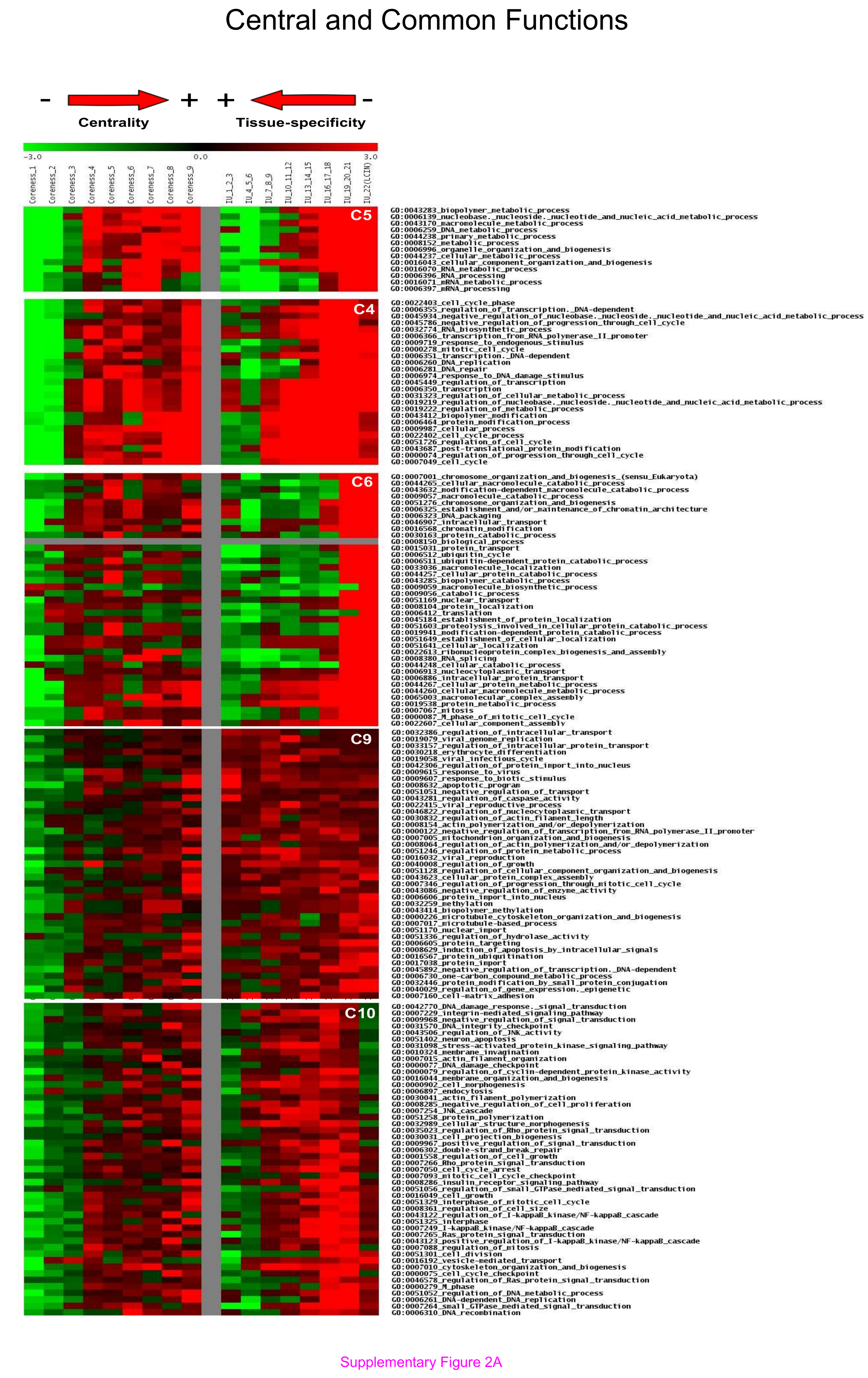

Supplement: Figure S4 — Central and common functions. K- core and interaction usage heatmaps for cluster 4, 5, 6, 9 and 10. (TIF) [file pone.0022051.s004.tif]

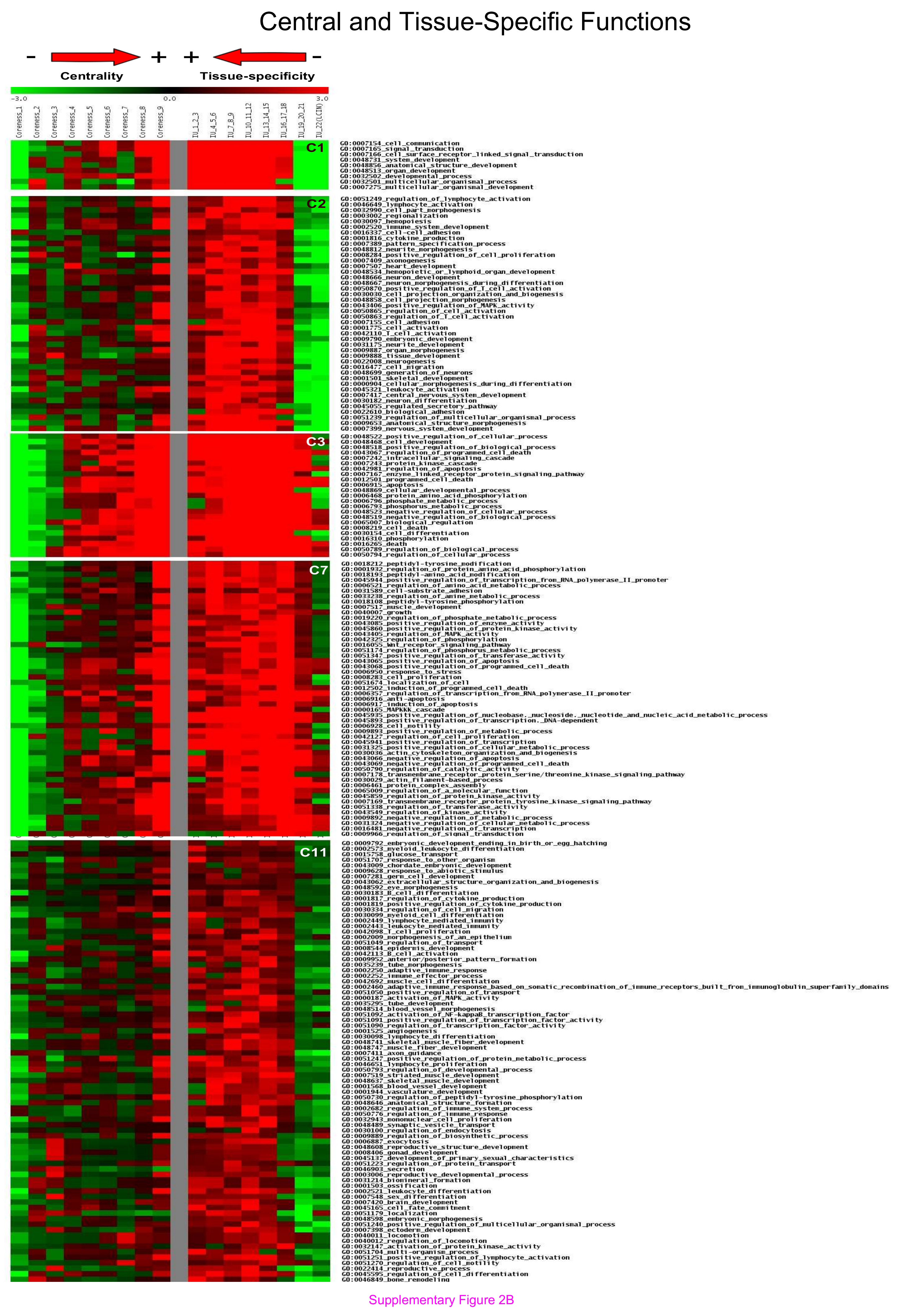

Supplement: Figure S5 — Central and tissue-specific functions. K-core and interaction usage heatmaps for cluster 1, 2, 3, 7 and 11. (TIF) [file pone.0022051.s005.tif]

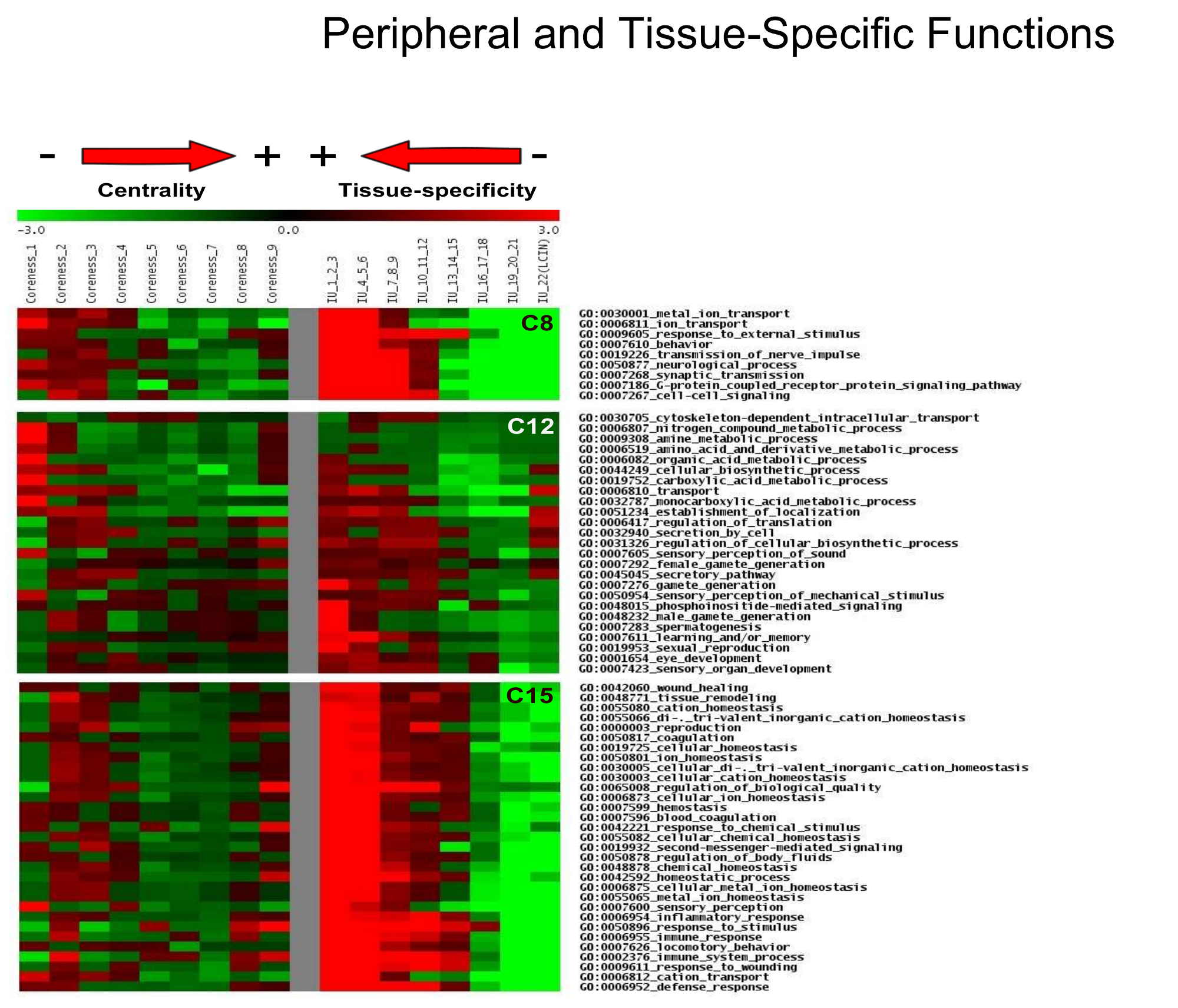

Supplement: Figure S6 — Peripheral and tissue-specific functions. K-core and interaction usage heatmaps for cluster 8, 12 and 15. (TIF) [file pone.0022051.s006.tif]
