# Supplementary material for: Functional Integrative Levels in the Human Interactome Recapitulate Organ Organization
Source: PLoS One. 2011 Jul 20;6(7):e22051. doi: 10.1371/journal.pone.0022051 (PMC3140469; doi:10.1371/journal.pone.0022051)
Supplement: File S1 — Choosing expression data to contextualize interactome: ESTs vs. Microarrays. (DOC) [file pone.0022051.s013.doc]

**Supplementary File 1**

**CHOOSING EXPRESSION DATA TO CONTEXTUALIZE INTERACTOME:**

**ESTs vs. MICROARRAYS.**

1. **Expression breath**

According to Zhu et al. [1], EST data are not saturated, therefore limiting gene detectability and increasing the number of false negative for tissue-specific genes. On another hand, due to the thresholds chosen to analyze microarray data, Zhu et al. show that those exhibit a high rate of false negative, leading to an underestimation of the number of housekeeping genes.

We verified these assumptions by building the distribution of the expression breadth (number of tissues in which the genes are expressed) from the microarray data compiled in Gene Expression Atlas (GXA) [2] and from the Unigene clusters of ESTs [3].

*Figure R1: Comparison of EST and microarray expression breadths. Magenta bars: percentage of genes expressed considering EST data. Blue bars: percentage of genes expressed considering microarray data.*

With an overall equivalent number of expressed genes (17247 and 17122 for GXA and ESTs, respectively), the proportion of transcripts detected in all tested tissues is larger with ESTs than microarrays (22,6% vs. 7,5% respectively), therefore confirming the lack of housekeeping genes detection in microarrays. However, the number of tissue-specifically expressed genes is also noticeably higher in ESTs (3,4%) than in microarrays (0,4%). This was not expected according to [1] and is probably due to the fact that the Unigene EST data used in this study are more saturated than those used in the original study [1].

1. **Benchmarking on immuno-detected proteins**
2. **Housekeeping proteins**

To further verify this depletion of housekeeping genes in microarray expression analysis, we compared expression data from EST and GXA to an orthogonal protein dataset, composed of proteins immuno-detected in different cell and tissue types, and compiled in the Human Protein Atlas [4].

Table R1 presents the results. Although the percentages of expressed genes according to GXA (72%), ESTs (69%) and to the proteins expressed in tissues (68%) are comparable, the numbers of genes/proteins expressed in all tissues are radically different. Whereas protein expression and ESTs behave similarly, representing 20% of the expressed proteins/genes, they only represent 7.5% of the genes expressed in all the investigated tissues according to GXA.

|  | **Human Protein Atlas** ^1^ | **ESTs**^2^ | **GXA**^3^ |
| --- | --- | --- | --- |
| **Nb investigated cell types / tissues** | 65 | 22 | 21 |
| **Method** | Immunological detections | Data integration, inference from EST expression | Inference from transcriptome experiments |
| **% expressed proteins**^1^**/ proteome**^2^ **(out of 4842**^1^**/ 17141 proteins**^2^ **/ 18176 genes**^3^**)** | 68 % | 69% | 72% |
| **Proteins commonly expressed (>60 cell types**^1^**/22 tissues**^2^ **/ 21 tissues**^3^**)** | 20% | 20% | 7.5% |
| **Proteins specifically expressed (<6 cell types**^1^**/ 1 tissue**^2, 3^**)** | 3% | 3,4% | 0.4% |

*Table R1: Comparison of protein data to both types of expression data, ESTs and microarrays. The second column (Human Protein Atlas) represents proteins immuno-detected in 65 cell and tissue types from Human Protein Atlas. The third column (ESTs) compiles results obtained when inferring protein presence based on EST expression. The fourth column (GXA) compiles results obtained when inferring protein presence based on GXA microarray data.*

**This suggests that microarray results lack a part of the widely expressed genes whereas ESTs are more comparable to the protein detection results.**

1. **Strictly tissue-specific proteins**

In a last comparison, we investigated the tissue-specific proteins identified in the Human Protein Atlas. We considered the 75 proteins found exclusively expressed in one tissue by immuno-detection, verified the breadth of the expression of their mRNA according to GXA and EST and plotted it. **We found a better accordance of the EST data with *bona fide* protein data than microarray data (see R2 plot legend for details). However, this result may be observed because ESTs are not saturated.**


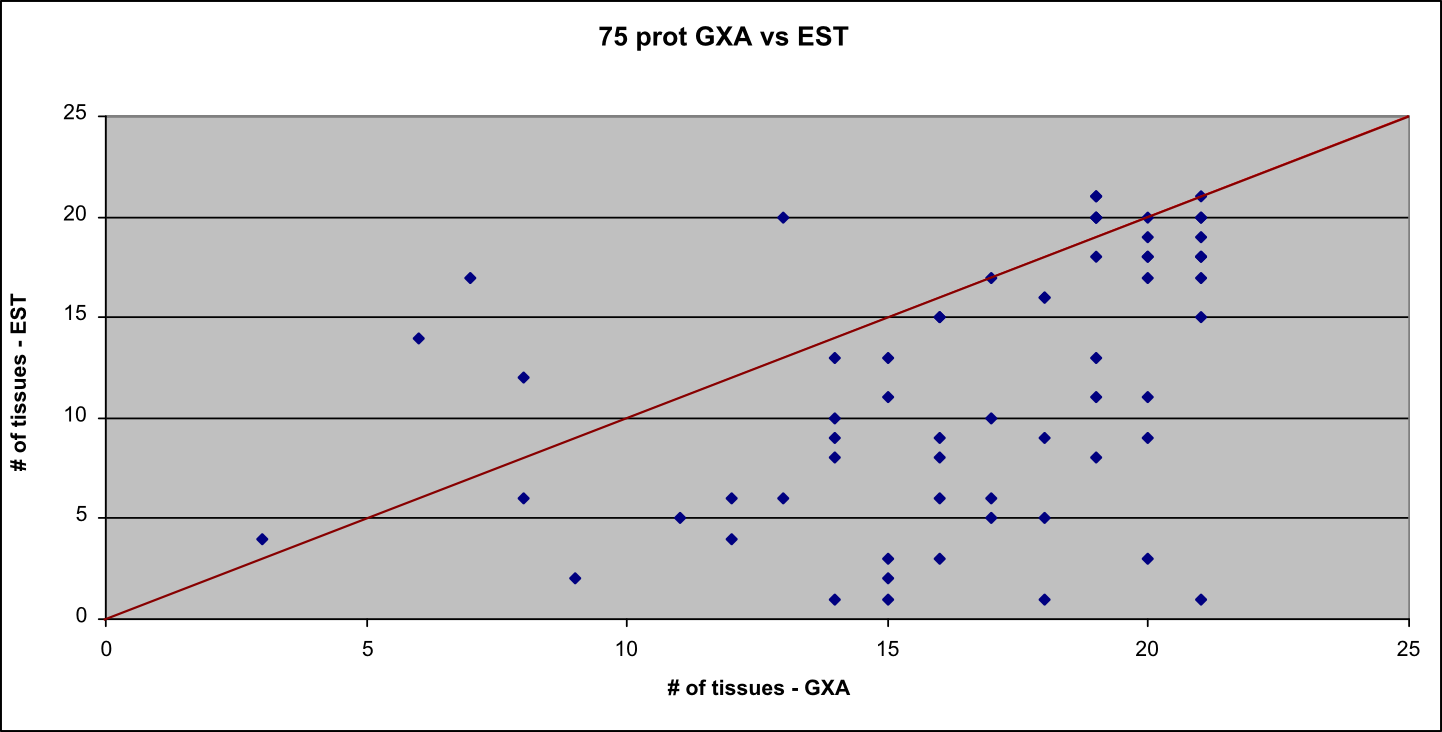


***Figure R2: In how many tissues do we infer the presence of the 75 tissue-specific proteins according to the Human Protein Atlas based on EST data (y-axis) and microarray GXA data (x-axis)? The red line represent the same number of inferred presences for both methods : a dot under the red line implies that the number of tissues where the protein is inferred is higher using GXA data, while a dot above the red line implies that the number of tissues where the protein is inferred is higher using EST data. Pink lines show the median values for each inference type (median = 11 and 17 for EST and GXA, respectively).***

1. Zhu J, He F, Song S, Wang J, Yu J (2008) How many human genes can be defined as housekeeping with current expression data? BMC Genomics 9: 172.

2. Kapushesky M, Emam I, Holloway E, Kurnosov P, Zorin A, et al. (2010) Gene expression atlas at the European bioinformatics institute. Nucleic Acids Res 38: D690-8.

3. Wheeler D, Church D, Federhen S, Lash A, Madden T, et al. (2003) Database resources of the National Center for Biotechnology. Nucleic Acids Res 31: 28-33.

4. Ponten F, Gry M, Fagerberg L, Lundberg E, Asplund A, et al. (2009) A global view of protein expression in human cells, tissues, and organs. Mol Syst Biol 5: 337.
